# Removal of MuRF1 Increases Muscle Mass in Nemaline Myopathy Models, but Does Not Provide Functional Benefits

**DOI:** 10.3390/ijms23158113

**Published:** 2022-07-23

**Authors:** Johan Lindqvist, Justin Kolb, Josine de Winter, Paola Tonino, Zaynab Hourani, Siegfried Labeit, Coen Ottenheijm, Henk Granzier

**Affiliations:** 1 Department of Cellular and Molecular Medicine, 1656 E Mabel Street, University of Arizona, Tucson, AZ 85724, USA; johanlindqvist@email.arizona.edu (J.L.); justinkolb@email.arizona.edu (J.K.); toninop@arizona.edu (P.T.); zaynabh@arizona.edu (Z.H.); c.ottenheijm@amsterdamumc.nl (C.O.); 2 Department of Physiology, Amsterdam UMC, Location VUMC, 1081 HV Amsterdam, The Netherlands; jm.dewinter@amsterdamumc.nl; 3 Research, Innovation and Impact Core Facilities Department, University of Arizona, Tucson, AZ 85721, USA; 4 Department of Integrative Pathophysiology, Medical Faculty Mannheim, University of Heidelberg, 68167 Mannheim, Germany; labeit@medma.de

**Keywords:** nemaline myopathy, nebulin, muscle atrophy, MuRF1, NEM2 and MAFbx

## Abstract

Nemaline myopathy (NM) is characterized by skeletal muscle weakness and atrophy. No curative treatments exist for this debilitating disease. NM is caused by mutations in proteins involved in thin-filament function, turnover, and maintenance. Mutations in nebulin, encoded by NEB, are the most common cause. Skeletal muscle atrophy is tightly linked to upregulation of MuRF1, an E3 ligase, that targets proteins for proteasome degradation. Here, we report a large increase in MuRF1 protein levels in both patients with nebulin-based NM, also named NEM2, and in mouse models of the disease. We hypothesized that knocking out MuRF1 in animal models of NM with muscle atrophy would ameliorate the muscle deficits. To test this, we crossed MuRF1 KO mice with two NEM2 mouse models, one with the typical form and the other with the severe form. The crosses were viable, and muscles were studied in mice at 3 months of life. Ultrastructural examination of gastrocnemius muscle lacking MuRF1 and with severe NM revealed a small increase in vacuoles, but no significant change in the myofibrillar fractional area. MuRF1 deficiency led to increased weights of various muscle types in the NM models. However, this increase in muscle size was not associated with increased in vivo or in vitro force production. We conclude that knocking out MuRF1 in NEM2 mice increases muscle size, but does not improve muscle function.

## 1. Introduction

Nebulin is a long slender sarcomeric protein that lies in the grooves between the two actin strands of the thin filament in skeletal muscle [1,2]. Nebulin’s size varies between different muscles and species, and ranges from ~600 to ~900 kDa [3,4]. The protein structure is highly repetitive, with ~30 amino acid-long modules that bind to the actin monomers. Throughout most of the length of nebulin, seven of these modules form super-repeats [1,4]. The troponin–tropomyosin regulatory complex also has a 1:7 stoichiometry to actin, and studies on cardiac thin filaments suggest that the troponin T-linker region crosses the groove of the two actin strands and localizes to the nebulin-containing region of the thin filament in skeletal muscle [2]. Nebulin has been found to stabilize the thin filament, regulate calcium sensitivity, and cross-bridge cycling kinetics, which, in turn, affects the number of myosin heads that interact with actin to produce force [5,6,7,8,9]. Furthermore, nebulin plays a crucial role in specifying the minimum length of the thin filament and the organization of the Z disk [10,11,12]. In human and mouse genomes, a single copy of the *NEB* gene, with 183 and 165 exons, respectively, contains the coding information for the nebulin protein [13]. Because of its large size, the *NEB* locus is frequently mutated and is associated with several myopathies. Mutations in the NEB gene are the most common cause of nemaline myopathy (NM), known as NEM2 [14]. Recessive nebulin mutations can also cause distal nebulin myopathy without nemaline rods, distal nemaline/cap myopathy, and core–rod myopathy, and have been associated with distal muscle weakness and atrophy [14,15,16,17].

NM is one of the most common of the nondystrophic congenital myopathies [18]. It is a heterogeneous disease characterized by varying degrees of skeletal muscle weakness [19,20]. Histologically, a diagnostic hallmark in muscle biopsies is rod-shaped protein aggregates, so-called nemaline rods, that are stained red with modified Gomori trichrome and appear as electron-dense material on electron micrographs [19]. In addition, because of the highly variable presence of nemaline rods in biopsies, DNA sequencing is increasingly assisting diagnosis. Clinically, patients with NM often have reduced muscle bulk due to muscle atrophy/hypotrophy, combined with varying degrees of muscle weakness with different muscles being unequally affected [21]. Mouse models have recently become available for NM that phenocopy most of the typical hallmarks of NM, including changes in muscle trophicity, with fast muscle typically usually being atrophic [22,23]. These models will be valuable for development of therapies for NM.

In general, skeletal muscle atrophy is a consequence of various stress conditions, such as immobilization, denervation, hind-limb suspension, mechanical ventilation, cancer cachexia, and other chronic diseases [24]. The two main protein-degradation pathways in skeletal muscle are the autophagic–lysosomal and ubiquitin–proteasome systems [25]. Ubiquitin is a highly conserved 76 amino acid-long polypeptide that is activated by an E1 enzyme, which transfers the ubiquitin to an E2 ubiquitin-conjugating enzyme. An E3 ligase then transfers the ubiquitin to the substrate. The ubiquitinated protein is subsequently degraded by the 26S proteasome [26]. The two canonical E3 ligases in skeletal muscles are MuRF1 and MAFbx, which have been found to be upregulated during skeletal muscle stress conditions [27,28,29,30,31,32,33]. Thus, MuRF1 has been proposed to be a therapeutic target in such secondary myopathies accompanied by muscle atrophy.

We hypothesized that preventing or limiting the muscle atrophy/hypotrophy could be a therapeutic strategy in NM to counteract muscle smallness and accompanying weakness. We focused on MuRF1, as we have previously found it upregulated in a mouse model with nebulin deficiency and severe NEM2, whereas MAFbx protein levels were unchanged [23]. Accordingly, we studied the expression of MuRF1 in patients with NM and found it highly upregulated. In order to explore the therapeutic potential of MuRF1 inhibition, we crossed MuRF1 KO mice with two mouse models of NEM2. The first model carries compound heterozygous mutations in the nebulin gene and phenocopies the most common form of the disease [22]. The second one is the conditional nebulin knockout model that is nebulin-deficient and mimics severe NM [23]. We examined the effect of ablating MuRF1 protein expression on muscle function and size in these models.

## 2. Results

### 2.1. MuRF1 Protein Expression in NEM2

**Figure 1:**
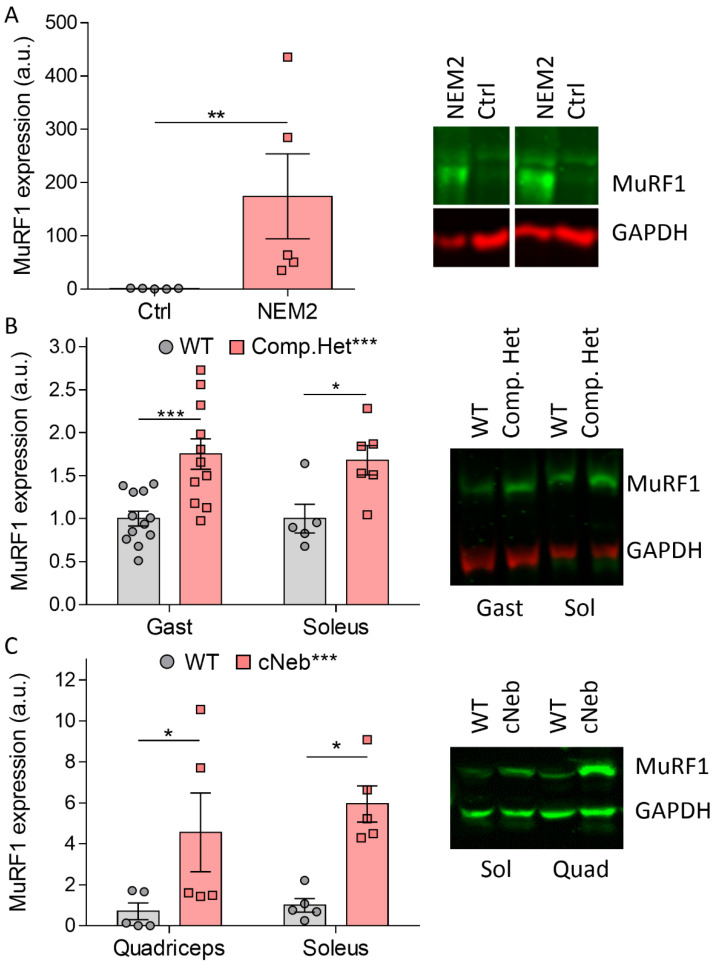
Expression of MuRF1 in nemaline myopathy patients and mouse models of nemaline myopathy. (**A**) MuRF1 expression levels in nebulin-based NM (NEM2) patients older than 5 years and representative Western blot image. Mann–Whitney’s ranked sum test was used for statistics. (**B**) Expression of MuRF1 in Compound-Het mice in gastrocnemius (Gast) and soleus (Sol) muscles with representative image to the right. (**C**) Expression of MuRF1 in cNeb mice in quadriceps (Quad) and soleus (Sol) muscles with representative image to the right. A two-way ANOVA with Sidak’s multiple-comparison test was used for statistical analysis for (**B**,**C**). Mixed sexes were used in the Western blots. * *p* < 0.05, ** *p* < 0.01 and *** *p* < 0.001.

The reduced muscle size associated with NM can be caused by excessive protein degradation, and thus we investigated the expression of MuRF1, a canonical protein involved in skeletal muscle atrophy. We studied MuRF1 protein levels in patients with NEM2 older than 5 years and in mouse models of typical and severe NM. The typical model is the Neb^S6366I^–Neb^Δexon55^ compound heterozygous mouse model (Compound-Het model) that we recently developed [22]. The severe model is the conditional nebulin knockout (cNeb) model with less than 10% of normal nebulin in adult muscle [23]. In biopsies from NEM2 patients, we found a very large (~170-fold) upregulation in MuRF1 protein expression compared to healthy controls. This large fold change was, in part, due to the low baseline level of MuRF1 in the controls (Figure 1A). Next, we examined the MuRF1 expression levels in the gastrocnemius and soleus muscles from 4-month-old Compound-Het mice. Both muscle types had a significant upregulation of MuRF1 protein levels (Figure 1B) compared to age-matched WT littermates. Interestingly, MuRF1 expression was significantly upregulated only in female Compound-Het gastrocnemius muscle when comparing MuRF1 protein levels between sexes with Sidak’s multiple comparison as post hoc test, although a highly significant genotype effect existed on two-way ANOVA using genotype and sex as factors (Supplemental Figure S1). Similarly, MuRF1 was significantly upregulated in both soleus and quadriceps muscles from 6-month-old cNeb mice compared to WT mice (Figure 1C). Taken together, these results indicate that MuRF1 is upregulated in NM and that this might contribute to the muscle smallness found in the disease.

To study whether inhibition of MuRF1 has therapeutic potential to counteract the muscle smallness found in NEM2, we crossed MuRF1 KO mice with Compound-Het and cNeb mice. These crosses were viable with a survival profile like that previously published for the Compound-Het and cNeb mice [22,23].

### 2.2. Body Weights

**Figure 2:**
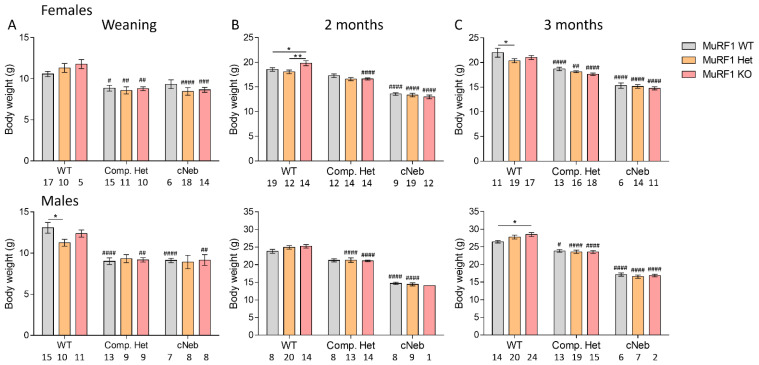
Body weights at different time points in two mouse models of nemaline myopathy. Top row shows female mice and bottom row shows male mice. (**A**) At weaning. (**B**) 2 months of life. (**C**) 3 months of life. Note the different axes between time points and sexes. The different colors indicate the MuRF1 genotype in crosses of healthy control (WT), typical (Comp. Het), and severe (cNeb) nemaline myopathy mouse models. Numbers below graphs indicate N-values. A two-way ANOVA with Tukey’s post hoc test was used for statistical testing. * Significant statistical difference vs. MuRF1 WT in that model; # significant statistical difference vs. healthy WT with similar MuRF1 genotype. * *p* < 0.05 and ** *p* < 0.01. # *p* < 0.05, ## *p* < 0.01, ### *p* < 0.001 and #### *p* < 0.0001.

We measured body weights for all crosses at weaning (~3 weeks), 2 months, and 3 months of life. As previously shown [22,23], Compound-Het and cNeb that are WT for MuRF1 have decreased body weights compared to MuRF1 WT control mice, except at 2 months of life. Loss of MuRF1 resulted in no large differences between any model at any time point. Small body weight differences were found in control mice lacking MuRF1, but no effects were detected in the Compound-Het or cNeb models (Figure 2).

### 2.3. Grip Strength

**Figure 3:**
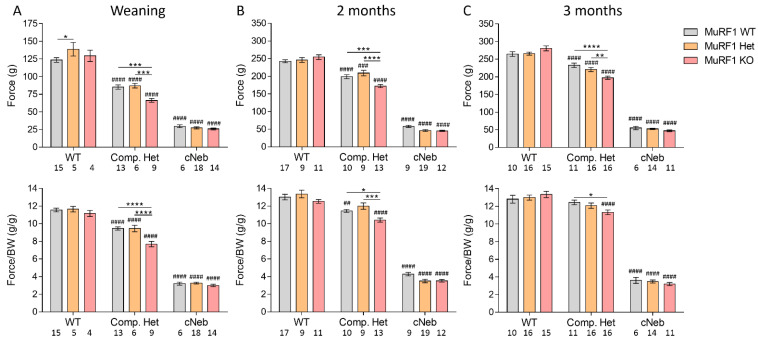
Four limb grip strength of female mice at different time points in two mouse models of nemaline myopathy. The top row shows absolute grip strength in grams of force. Bottom row shows grip strength normalized to body weight. (**A**) At weaning (left panel). (**B**) 2 months of life (middle panel). (**C**) 3 months of life (right panel). Note the different Y-axis between different time points. The different colors indicate MuRF1 genotype in crosses of healthy control (WT), typical (Comp. Het) and severe (cNeb) nemaline myopathy mouse models. Numbers below graphs indicate N-values. A two-way ANOVA with Tukey’s post hoc test was used for statistical testing. * indicates significant statistical difference vs. MuRF1 WT in that model. # indicates significant statistical difference vs. healthy controls with similar MuRF1 genotype. * *p* < 0.05, ** *p* < 0.01, *** *p* < 0.001 and **** *p* < 0.0001. ## *p* < 0.01, ### *p* < 0.001 and #### *p* < 0.0001.

Grip strength was used to determine in vivo voluntary muscle function at weaning, 2 months, and 3 months of life. Figure 3 (top) shows the absolute grip strength and Figure 3 (bottom) the body-weight-normalized grip strength in female mice. As expected, at all three ages, absolute grip strength was lower in Compound-Het and cNeb mice than healthy controls. Body-weight-normalized grip strength was lower in cNeb mice at all three ages, but it was only lower for Compound-Het at 3 weeks of life compared to control mice. MuRF1 deficiency reduced both absolute and body-weight-normalized grip strength in Compound-Het mice, but did not affect cNeb mice (Figure 3). Similar findings were obtained in male mice (Supplemental Figure S2).

### 2.4. Tissue Weights

**Figure 4:**
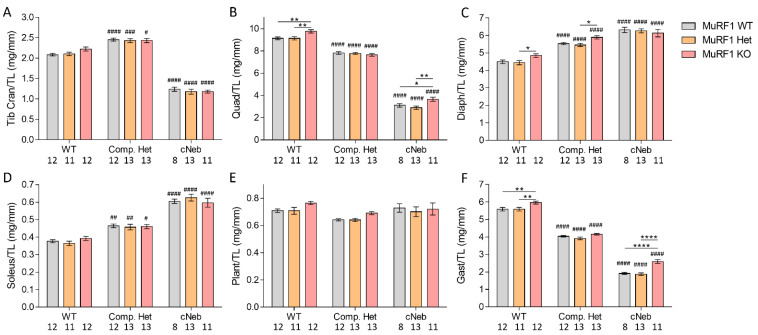
Muscle weights normalized to tibia length for female mice at 3 months of life. (**A**) Tibialis Cranialis. (**B**) Quadriceps. (**C**) Diaphragm. (**D**) Soleus. (**E**) Plantaris. (**F**) Gastrocnemius. The different colors indicate MuRF1 genotype in crosses of healthy control (WT), typical (Comp. Het) and severe (cNeb) nemaline myopathy mouse models. Numbers below graphs indicate N-values. A two-way ANOVA with Tukey’s post hoc test was used for statistical testing. * indicates significant statistical difference vs. MuRF1 WT in that model. # indicates significant statistical difference vs. healthy WT with similar MuRF1 genotype. * *p* < 0.05, ** *p* < 0.01 and **** *p* < 0.0001. # *p* < 0.05, ## *p* < 0.01, ### *p* < 0.001 and #### *p* < 0.0001.

At 3 months of life, multiple muscle types were dissected, and weights were recorded and normalized to the tibia lengths. Figure 4 shows results from female mice. MuRF1 deficiency did not affect the weight of some muscle types and increased the weight of others. For example, MuRF1 deficiency in female cNeb mice increased the quadriceps weight by 18% and the gastrocnemius weight by 35% (Figure 4B,F). Similar findings were made in male mice (Supplemental Figure S3). The differences between healthy WT and NEM2 mice are similar to the results from our initial publications of these models, including the increased muscle weights of the soleus muscle that we previously showed was due to an increase in the number of fibers, whereas individual soleus fibers were atrophied [22,23].

### 2.5. In Vivo and In Vitro Muscle Mechanics

**Figure 5:**
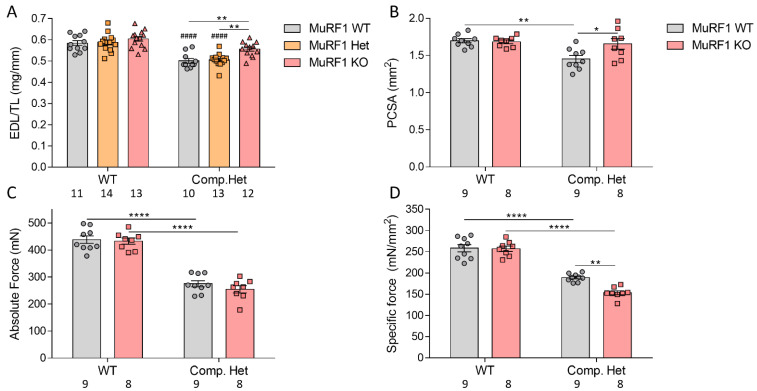
Intact mechanics of EDL muscle in Compound-Het male mice. (**A**) EDL muscle weights normalized to tibia lengths. (**B**) Physiological cross-sectional area (PCSA). (**C**) Absolute force. (**D**) Specific force (force normalized to PCSA). Numbers below graphs indicate N-values. A two-way ANOVA with Tukey’s post hoc test was used for statistical testing. * Significant statistical difference vs. MuRF1 WT in that model. # Significant statistical difference vs. healthy WT with similar MuRF1 genotype. * *p* < 0.05, ** *p* < 0.01 and **** *p* < 0.0001. #### *p* < 0.0001.

**Figure 6:**
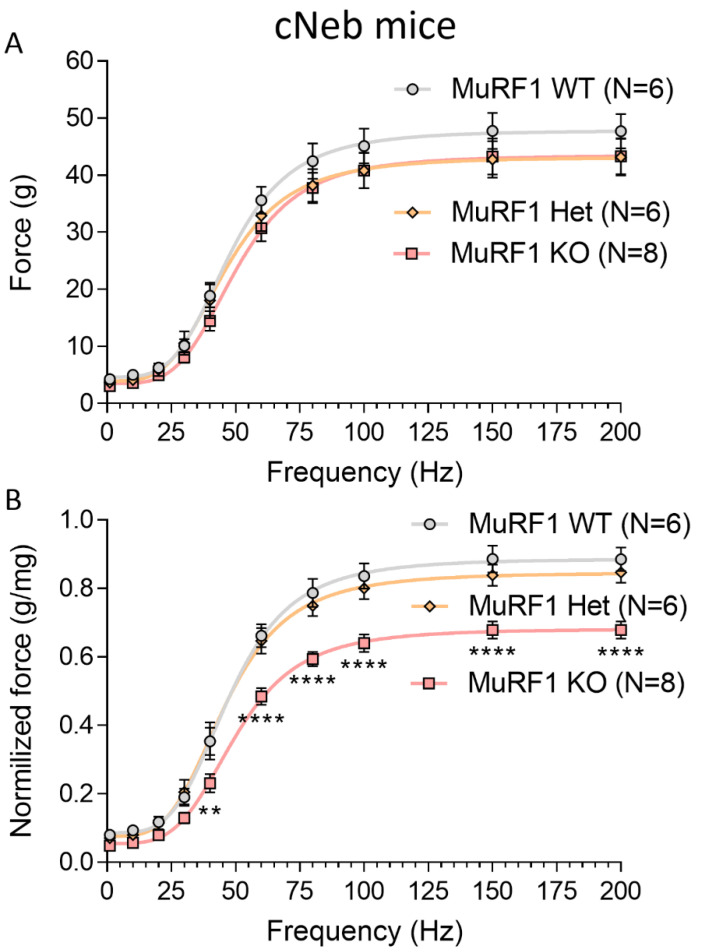
Force production of the lower back limb muscles determined by an in vivo foot-plate system in female MuRF1-deficient conditional Nebulin knockout mice. (**A**) Absolute force. (**B**) Force normalized to gastrocnemius complex tissue weight. A two-way ANOVA with Tukey’s post hoc test was used for statistical testing. ** *p* < 0.01 and **** *p* < 0.0001.

To study whether the increased weights of MuRF1 deficient muscle results in increased force production, we initially studied in vitro muscle function. EDL muscles from 3-months old WT control and Compound-Het male mice were used and the force-frequency relation at optimal muscle length was measured. MuRF1 deficiency resulted in increased muscle weights and larger cross-sectional areas in male Compound-Het (Figure 5A,B). MuRF1 deficiency in WT control mice did not affect the absolute force or specific force (Figure 5C,D). In Compound-Het mice, knocking out MuRF1 resulted in no change in absolute force and a 20% decrease in specific force (Figure 5C,D). We also investigated the function of the gastrocnemius muscle in female cNeb mice (where this muscle type is increased in weight (Figure 4F) and used for this an in vivo foot plate system. Absolute gastrocnemius force production was unaffected by MuRF1 in cNeb mice (Figure 6A) whereas muscle weight normalized force was reduced at a wide range of stimulation frequencies, e.g., by 35% at a stimulation frequency of 200 Hz (Figure 6B).

### 2.6. Ultrastructural Studies on cNeb Gastrocnemius

**Figure 7:**
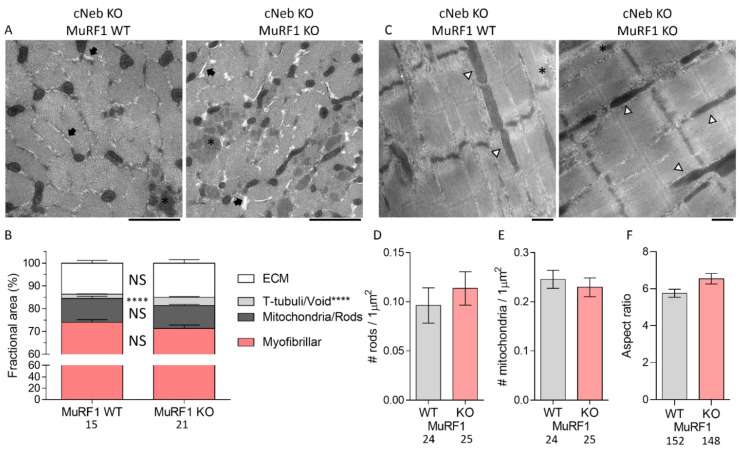
Electron micrographs of gastrocnemius muscles from conditional nebulin KO crossed with MURF1 KO mice. (**A**) Representative cross-sectional electron micrographs. Scale bars: 1 μm. (**B**) Quantified fractional cross-sectional area of compartments. (**C**) Representative longitudinal electron micrographs. Scale bars: 500 nm. (**D**) Number of nemaline rods per square micrometer. (**E**) Number of mitochondria per square micrometer. (**F**) Aspect ratio (length divided by width) of mitochondria. Asterisks show nemaline rod bodies. Arrows indicate T-tubules and void areas. Arrowheads point to mitochondria. Arrow heads indicate mitochondria. Numbers indicate number of analyzed images (mitochondria in (**F**)) from two mice for each genotype. Hierarchical t-tests were used for statistical testing **** *p* < 0.0001.

To gain insights into why the increase in gastrocnemius muscle mass did not increase muscle force, we performed ultrastructural studies on cross-sections of the proximal head of gastrocnemius muscles in MuRF1 WT and MuRF1 KO cNeb mice (Figure 7). We performed quantitative analysis of extracellular matrix, dark-stained electron-dense mitochondria, nemaline rods, and T-tubules/vacuoles (including areas devoid of other material) (Figure 7A). From these measurements, we calculated the fractional myofibrillar area. The area covered by T-tubules/vacuoles was slightly but significantly increased from 2% to 4% of total intracellular cross-sectional area in MuRF1-deficient gastrocnemius muscles compared to control cNeb muscles. There was no significant difference in the areas covered by mitochondria/nemaline rods or ECM. Myofibrillar content was also not different between MuRF1 WT and MuRF1-deficient cNeb gastrocnemius muscles (Figure 7B). We also examined the ultrastructure of longitudinal sections from the same muscles and quantified the number and length of nemaline rod bodies, mitochondria, and myofibril diameter (Figure 7C). The myofibril diameter at the M-line and nemaline rod body length across the Z-disk were normalized to sarcomere length. No differences in rod body or mitochondria numbers were found (Figure 7D,E). The mitochondria aspect ratio (mitochondria length divided by width) was not different (Figure 7F), but visually the mitochondria in cNeb MuRF1-deficient muscles appeared darker and had less distinct cristae (Figure 7C). Further, we did not find any differences in the nemaline rod body length or myofibril diameter in MuRF1 KO cNeb compared to MuRF1 WT cNeb mice (Supplemental Figure S4).

### 2.7. MAFbx Protein Expression

**Figure 8:**
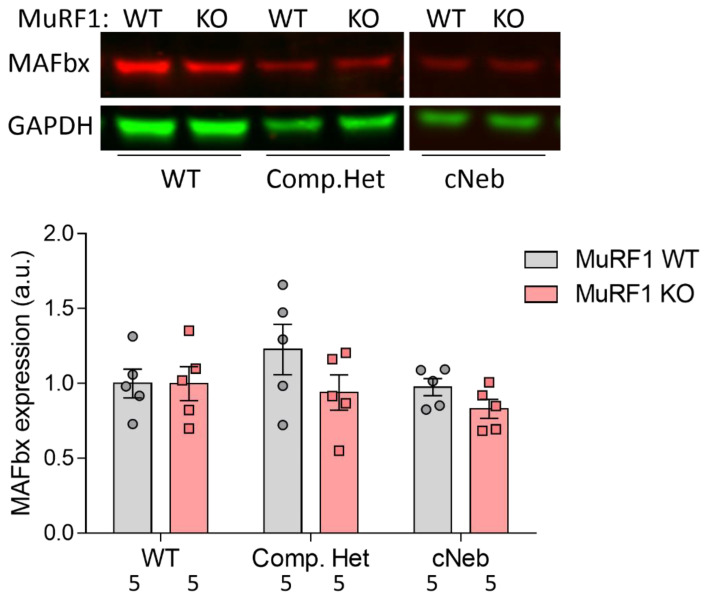
Expression of MAFbx protein in Compound-Het and cNeb mice. Top: Representative images of western blots. Bottom: Quantification of MAFbx protein levels in soleus muscles. Numbers below graph indicate N-values. A two-way ANOVA with Sidak’s post hoc test was used for statistical testing.

Finally, we investigated how MuRF1 deficiency affected protein levels of MAFbx, the other classical E3 ligase found in striated muscle. We performed Western blotting on soleus muscles from Compound-Het and cNeb mice (mixed sexes). No statistically significant changes in MAFbx expression were observed between MuRF1 WT Compound-Het, cNeb, and control mice. Furthermore, no changes in MAFbx expression were observed in Compound-Het and cNeb mice lacking MuRF1 compared to MuRF1 WT Compound-Het and cNeb mice, respectively (Figure 8).

## 3. Discussion

NM is characterized by muscle weakness and diminished muscle size due to muscle atrophy. Both of these characteristics have also been observed in animal models with mutations in NEB or ACTA1 (encodes skeletal muscle α-actin) [22,23,34,35,36]. The molecular mechanism underlying the muscle weakness is likely to include alterations in the contraction mechanism due to mutations in the sarcomeric proteins that disrupt the normal cross-bridge behavior [6,37,38,39,40,41]. However, the mechanisms underlying the muscle smallness remain obscure. In this study, we show in two NEM2 mouse models and in patients with NEM2 a large upregulation of the canonical striated muscle E3 ligase MuRF1. To our knowledge, this is the first time an upregulation of MuRF1 protein has been reported in human NM-patients. It is well established that MuRF1 is a key mediator of muscle atrophy during various acquired and chronic conditions and diseases [27,42]. Thus, the upregulation of MuRF1 that we found in both NM patients and NM mouse models could be a contributing factor to the muscle smallness in NM, possibly due to increased protein degradation. As of today, there are no approved therapies for NM. Considering the large upregulation of MuRF1 expression in patients with NEM2 and that nebulin mutations are the most common cause of NM [14], we tested whether inhibition of MuRF1 is a therapeutic option for NM. We knocked out MuRF1 in two mouse models of NEM2, one with the typical and the other with the severe form of the disease, and found that muscle weights were increased in select muscle types. Functional studies focusing on these select muscles indicated that this muscle weight increase was not associated with an increased force production. Below, we discuss these findings in detail.

The mechanism that underlies the increased muscle weight that was found in some of the studied muscles upon MuRF1 deletion was not studied, but several possibilities exist. A recently proposed model implicates MuRF1 ubiquitination of the titin kinase (TK) by binding to the A168–170 domains, localized at the N-terminal end of the TK [43,44,45]. In this model, ubiquitination of the TK by MuRF1 promotes the recruitment of p62 and its interacting partner Nbr1 to the M-band. P62 and Nbr1 are adaptor molecules that assemble onto polyubiquitinated proteins and promote their removal by selective autophagy [45]. Importantly, this process appears to be inhibited by muscle contraction, as this results in high forces on the M-band that unfold the N-terminal extension of the TK, increasing the distance between the MuRF1 binding site and its target sites on TK, thereby reducing or preventing TK ubiquitination and reducing the capability of TK to recruit Nbr1 and p62 [45]. The low forces generated by nebulin-deficient muscle are thus predicted to result in more MuRF1-dependent protein degradation and, consequently, deleting MuRF1 will block this process and increase muscle mass. An alternative or additional mechanism could be that MuRF1 acts as a brake on de novo muscle protein translation and loss of MuRF1 protein results in augmented protein synthesis and muscle growth [46]. Finally, the finding that not all muscle types are equally affected by MuRF1 deletion, for example, no effect was found in soleus muscles and a significant increase was present in EDL muscle, might at least in part be explained by the distinct fiber-type composition of these muscle types (soleus: rich in type I; EDL rich in type IIB) and the earlier finding that suggests that MuRF1 plays a more prominent role in fast type II fibers [32].

Despite the increased mass of the EDL muscle in MuRF1-deficient Compound-Het male and gastrocnemius muscles in female MuRF1-deficient cNeb mice, unexpectedly, total force was unaffected by deleting MuRF1 and normalized force of these muscles were reduced. One possible reason could be that knocking out MuRF1 results in a reduction in the myofibrillar fractional area. However, the ultrastructural analysis did not reveal any changes in the myofibrillar fractional area when comparing MuRF1-deficient cNeb muscle with MuRF1 WT cNeb muscle, making this explanation unlikely. Another possibility to consider is that the loss of MuRF1 activity leads to less protein ubiquitination and a decrease in the rate of protein degradation, the likely source of the increased muscle size in MuRF1-deficient NM muscle, and that this causes an accumulation of deleterious posttranslational modifications of sarcomeric proteins, such as advanced glycation end products or oxidation. Both advanced glycation end products and oxidative nitration modifications have been reported to negatively affect striated muscle function [47,48,49]. In this scenario, the myofibrillar fractional cross-sectional area is maintained, as we observed, but each myofibril produces less force. The force deficit per myofibril would counteract the increased total area of myofibrils and could explain why MuRF1 KO Compound-Het and cNeb mice have a decreased in vivo muscle function, as seen in grip-strength and foot-plate experiments. The possibility of accumulation of advanced glycation end products is supported by the fact that MuRF1 is involved in glucose metabolism [50] and that our ultrastructural studies revealed darker mitochondria with less well-organized cristae. Clearly future mass spectroscopy studies are warranted to confirm whether there is an accumulation of advanced glycosylation products or other deleterious posttranslational modifications in MuRF1-deficient NM muscle.

We also investigated the expression of MAFbx, the other main E3 ligase found in striated muscle, as it is possible that the lack of MuRF1 affects the expression of other E3 ligases. However, we found no difference in MAFbx protein levels between Compound-Het and control mice, regardless of their MuRF1 genotype. This is consistent with the earlier finding that MAFbx was not upregulated in cNeb mice [23]. Thus, it appears that MAFbx plays a lesser role in NM than MuRF1. This is different from studies on other diseases and atrophy-causing conditions. For example, in a study on the effect of denervation in MuRF1 KO mice, higher MAFbx mRNA levels were found compared to WT mice [51]. Another study showed that muscle atrophy induced by caloric restriction caused increased expression of MAFbx mRNA, but this increase was diminished in MuRF1 KO mice [52]. Ubiquitination is also associated with DNA repair, endocytosis, kinase activation, signal transduction, and gene expression [53,54]. These studies show the complex biology of E3 ligases, ubiquitination, and the type of atrophy-inducing condition. More studies are needed to understand how these factors contribute to the muscle atrophy seen in NM.

Finally, it is important to discuss the recent discovery of small-molecule inhibitors that downregulate MuRF1 function by interfering with its recognition of titin A168–170 domains [55,56]. In multiple animal studies of muscle disease (cachexia, myocardial infarction, or heart failure with preserved ejection fraction), these small-molecule compounds were shown to protect muscles from wasting [55,56]. Like in our study, effects were limited or absent in muscle types that express slow type I fiber types (e.g., soleus), but an increase in muscle size was present in fast type II muscle types (e.g., EDL) [56]. However, unlike in the present study, EDL muscles from inhibitor-treated animals produced higher forces at both the absolute force and the specific force levels [56]. A possible explanation could be that the pharmacological targeting of MuRF1 was started in adult animals where the musculature was mature and under those conditions a positive effect on muscle function can be found. In contrast, constitutively knocking out MuRF1, like we did, will also affect embryonic, fetal, and early postnatal functions of MuRF1 and this might negate beneficial functional effects. Future pharmacological MuRF1 inhibition studies in NEM2 models that start in adult mice are warranted.

Study limitations: The number of available biopsies was limited and the biopsies were acquired for diagnostic purposes that determined the choice of muscle type. This explains the incomplete age, sex, and muscle matching between the patient and control samples that were used to determine MuRF1 protein levels in NEM2 patients. We limited our MuRF1 Western blots to samples from patients older than 5 years to reduce the influence of early postnatal skeletal muscle development. Analyzing MuRF1 expression in Compound-Het mice using a two-way ANOVA with genotype and sex as factors revealed that genotype was a significant source of variance, while sex was not. However, post hoc testing showed that MuRF1 was only upregulated in female Compound-Het mice. There is limited evidence for sex-dependent phenotypes in NM. One mouse model of actin-based NM has increased mortality rates in males compared to females [57], likely due to urethral obstruction resulting in bladder distension, inflammation, and necrosis in the males [58]. In the same model, the contractile deficits in female limb muscles are less severe than in males [59]. Future studies should examine the contractile phenotype and MuRF1 expression level in a wide range of muscle types and in both sexes. Further, not all NEM2 mouse muscles had reduced mass, for example, soleus and diaphragm muscles were larger in Compound-Het and cNeb mice than healthy WT mice. We have previously shown that the increased soleus weight is due to an increased number of muscle fibers with decreased cross-sectional area (CSA) [22,23]. This could possibly be related to the relatively high number of type I fibers in these muscles. The decreased CSA of individual soleus fibers is consistent with MuRF1 regulating skeletal muscle atrophy. How MuRF1 deficiency affects the muscle-fiber cross-sectional area and connective tissue content in the models used in this study should be studied in follow-up work.

In summary, we report increased MuRF1 expression in NEM2 patients and mouse models with NEM2. Crossing MuRF1 knockout mice to established NEM2 mouse models revealed that MuRF1 deficiency enlarges some fast-twitch muscles in both NEM2 and healthy WT mice. This increase was not associated with improved muscle-force production of NEM2 muscles, indicating that no functional benefit was obtained from increasing muscle mass.

## 4. Materials and Methods

### 4.1. Human Subjects

The study of human biopsies was approved under STUDY00000249 by the Institutional Review Board at the University of Arizona. Table S1 shows the characteristics of biopsy donors used for MuRF1 Western blots.

### 4.2. Animals

The different mouse models have previously been described [22,23,46], i.e., the model for *NEB* compound heterozygosity (one of the NEB alleles has a point mutation corresponding to nebulin Ser6366Ile found in humans, while the other allele has an exon 55 deletion; Compound-Het) with a phenotype resembling typical NM [22], the conditional nebulin knockout (cNeb) model for severe homozygous NM [23], and the MuRF1 gene inactivation (knockout) model for MuRF1 deficiency [46]. MuRF1 KO mice were crossed with Compound-Het and cNeb mice. The litters from the Compound-Het mice were heterozygous for either Neb^S6366I^ and MuRF1 or heterozygous for Neb^ΔExon55^ and MuRF1. The offspring were crossed to create Neb^S6366I^–Neb^Δexon55^ Compound-Het mice, while being wild-type, heterozygous, or KO for MuRF1. In a similar fashion, homozygous cNeb mice deficient in MuRF1 were created. All mouse lines were on C57/Bl6J backgrounds. Table S2 shows the genotypes of the different groups that were studied. Mice were housed at the animal care facility at the University of Arizona. The mice had food and water ad libitum and the room was maintained on 14:10 h light/dark cycles. The study was approved by the IACUC at the University of Arizona.

At 3 months of life, the mice were euthanized by isoflurane anesthesia and killed by cervical dislocation. The following muscles were isolated and weighed: tibialis cranialis (Tib Cran), extensor digitorum longus (EDL), quadriceps (Quad), gastrocnemius (Gast), plantaris (Plant), soleus, and diaphragm (Diaph). The muscle weights were normalized to the tibia lengths.

### 4.3. Western Blotting

Muscle samples were prepared following a well-documented protocol [60]. Tissues were pulverized to powder via glass Dounce homogenizers prechilled in liquid nitrogen. Tissue powder was allowed to equilibrate at −20 °C for 20 min before a 50% glycerol/H_2_O solution with protease inhibitors (in mM: 0.04 × 10^−64^, 0.16 leupeptin and 0.5 PMSF) and a urea buffer (in M: 8 urea, 2 thiourea, 0.050 tris–HCl, 0.075 dithiothreitol, 3% SDS *w*/*v* and 0.03% bromophenol blue, pH of 6.8) were added in a 1:40:40, sample (mg):glycerol (μL):urea (μL) ratio. The solution was mixed and incubated at 60 °C for 10 min before being aliquoted and flash-frozen in liquid nitrogen. For Western blotting, solubilized samples were run on a 10% polyacrylamide gel and transferred onto polyvinylidene difluoride membranes using a semidry transfer unit (Trans-Blot Cell, Bio-Rad, Hercules, CA, USA). Blots were stained with Ponceau S to visualize the total protein transferred. Blocking, detection with infrared fluorophore-conjugated secondary antibodies, and scanning followed recommendations for the Odyssey Infrared Imaging System (LI-COR Biosciences, Lincoln, NE, USA). The same muscle lysate from one C57Bl6J mouse was used in each Western blot to facilitate comparison between blots. The following primary antibodies were used for Western blotting: anti-MuRF1 (1:1000, chicken polyclonal, 11005, Myomedix, Neckargemünd, Germany) and MAFbx (1:1000, rabbit monoclonal recombinant, ab168372, Abcam, Cambridge, UK). Protein expression was normalized to glyceraldehyde-3-phosphate dehydrogenase (GAPDH, 1:5000, mouse monoclonal, MAS-15738, Invitrogen, Waltham, MA, USA). Gastrocnemius and soleus muscles from male and female Compound-Het and cNeb mice were used.

### 4.4. Grip Strength

All four limb grip-strength measurements were performed according to Tinklenberg et al. [58]. The mouse was placed on a horizontal steel mesh while the experimenter was holding its tail and allowed to pull away from the experimenter. Peak tensions (grams of force) from the pull were recorded on a digital force gauge (Chatillon Force Measurement DFEII, Columbus Instruments, Columbus, OH, USA). The mice were tested after weaning (~23 days of life), and at 2 and 3 months of life.

### 4.5. Body Weights

Body weights were collected at the time of grip-strength measurements.

### 4.6. In Vivo Foot-Plate Experiments

In vivo muscle analysis for the gastrocnemius complex was conducted using a previously described protocol [22]. Three-month-old cNeb mice were anesthetized using isoflurane and placed on the heated platform (39 °C) of the Aurora Scientific Mouse Muscle Physiology System (model 809B; Aurora Scientific Inc., Aurora, ON, Canada). Hair was removed from the right hind-leg and the knee immobilized using a noninvasive clamp. The foot was secured to the footplate on the force transducer (300C series with dual-mode lever systems, Aurora Scientific) with adhesive tape and set at a 90° angle. Needle electrodes were placed distal to the knee, just under the skin in close proximity to the tibial and sural nerves. Optimal needle placement and pulse phase for plantar flexion was established using 10 Hz tetanus stimulations at 40 mA. Forces were measured in mN using ASI 610A Dynamic Muscle Control 5.3 software (Aurora Scientific Inc., Aurora, ON, Canada). Optimal current was determined using twitch forces measured every 10 s. The isometric force-frequency relationship was measured at 1, 10, 20, 30, 40, 60, 80, 100, 125, 150, and 200 Hz using the same stimulation parameters as described for 10 Hz stimulations (see force-frequency sequence below). Maximum tetanic force was typically achieved at 150 Hz. Tissue weights for the gastrocnemius complex (gastrocnemius, plantaris, and soleus) were used for force normalization.

### 4.7. Intact Muscle Mechanics

The intact muscle mechanics have been described previously [22,33,34]. In short, EDL muscles from 3-month-old Compound-Het and WT mice were carefully, but quickly, excised and silk suture loops (USP 4–0) were tied to each tendon. The muscle was attached to a stationary hook and a servomotor-force transducer connected to an Aurora Scientific 1200A isolated muscle system, and muscles were submerged in an oxygenated Krebs–Ringer bicarbonate solution at 30 °C (in mM: 137 NaCl, 5 KCl, 1 NaH_2_PO_4_·H_2_O, 24 NaHCO_3_, 2 CaCl_2_·2H_2_O, 1 MgSO_4_·7H_2_O and 11 glucose; pH 7.4). Optimal length (L0) was found by first performing a tetanus to remove any slack in the sutures, allowing the muscle to recover, and then increasing length until twitch forces plateaued. Force-frequency relation was determined by subjecting muscles to increasing stimulation frequencies (in Hz: 1, 10, 20, 40, 60, 80, 100, 150 and 200). Muscles were allowed to recover for 30, 30, 60, 90, 120, 120, 120 and 120 s between subsequent stimulations. Force obtained (converted to mN) was normalized to the physiological cross-sectional area (PCSA) through the following equation: PCSA = mass(mg)/[muscle density (mg/mm^3^) × fiber length (mm)]. The physiological density of muscle is 1.056 mg/mm^3^ and fiber length was found utilizing a fiber length to muscle length ratio of 0.51 for EDL [61].

### 4.8. Transmission Electron Microscopy

For ultrastructural cross-sectional analysis, we utilized a technique previously published [5,22]. The proximal head of the gastrocnemius muscle was fixed in a mixture of 3.7% paraformaldehyde, 3% glutaraldehyde, and 0.2% tannic acid in 10 mM PBS, pH 7.2 at 4 °C for 1 h. The muscles were then rinsed for 15 min in PBS and postfixation performed in 1% OsO4 in the same buffer for 30 min. Subsequently, samples were dehydrated in an ethanol graded series, infiltrated with propylene oxide and transferred to a mixture of 1:1 propyleneoxide:Araldite 502/Embed 812 resin (Epon-812, EMS), then to a pure Araldite 502/Embed 812 resin, and finally polymerized for 48 h at 60 °C. Longitudinal ultrathin sections (80 nm) were also obtained with a diamond knife (Diatome) in a Reichert Jung ultramicrotome and contrasted with 1% potassium permanganate and lead citrate. Images (1792 × 1792 pixels) were acquired in a Tecnai Spirit G2 transmission electron microscope (FEI, Hillsboro, OR, USA) with a side-mounted AMT Image Capture Engine 6.02 (4 Mpix) digital camera, operated at 100 kV. CellProfiler (version 2.2.0) (Broad Institute of MIT and Harvard, Boston, MA, USA) with custom scripts and Fiji ImageJ2 (version 1.53q) (University of Wisconsin, Madison, WI, USA) were used for image analysis.

### 4.9. Statistical Analysis

Data are represented as means ± SEM. GraphPad Prism (version 6.07) (GraphPad Software, San Diego, CA, USA) was used for statistical testing and generation of graphs. N-values are indicated in the figures. Statistical comparisons were restricted to groups with same sex, except for Western blot experiments. No comparison was made between sexes, unless noted otherwise. Rout’s outlier test (1% cutoff) was used to detect outliers. We used two-way ANOVA with multiple-comparison correction for MuRF1 genotype and Tukey’s or Sidak’s post hoc test as indicated. In a separate statistical test, we compared all groups using two-way ANOVA and Tukey’s post hoc test. For Figure 1A, a nonparametric Mann–Whitney test was used. Hierarchical *t*-test were used in Figure 7B–F and Supplemental Figure S4 (GraphPad Prism (here we used version 9.3.1)).

## Data Availability

The data presented in this study are available on request from the corresponding author.

## Supplementary Materials

The following supporting information can be downloaded at: https://www.mdpi.com/article/10.3390/ijms23158113/s1. References [62,63] are cited in the supplementary materials.
