# Supplementary material for: Removal of MuRF1 Increases Muscle Mass in Nemaline Myopathy Models, but Does Not Provide Functional Benefits
Source: Int J Mol Sci. 2022 Jul 23;23(15):8113. doi: 10.3390/ijms23158113 (PMC9331820; doi:10.3390/ijms23158113)
Supplement: Supplementary file 1 [file ijms-23-08113-s001.zip › Supplemental Material 2022-06-27.pptx]

## Slide 1
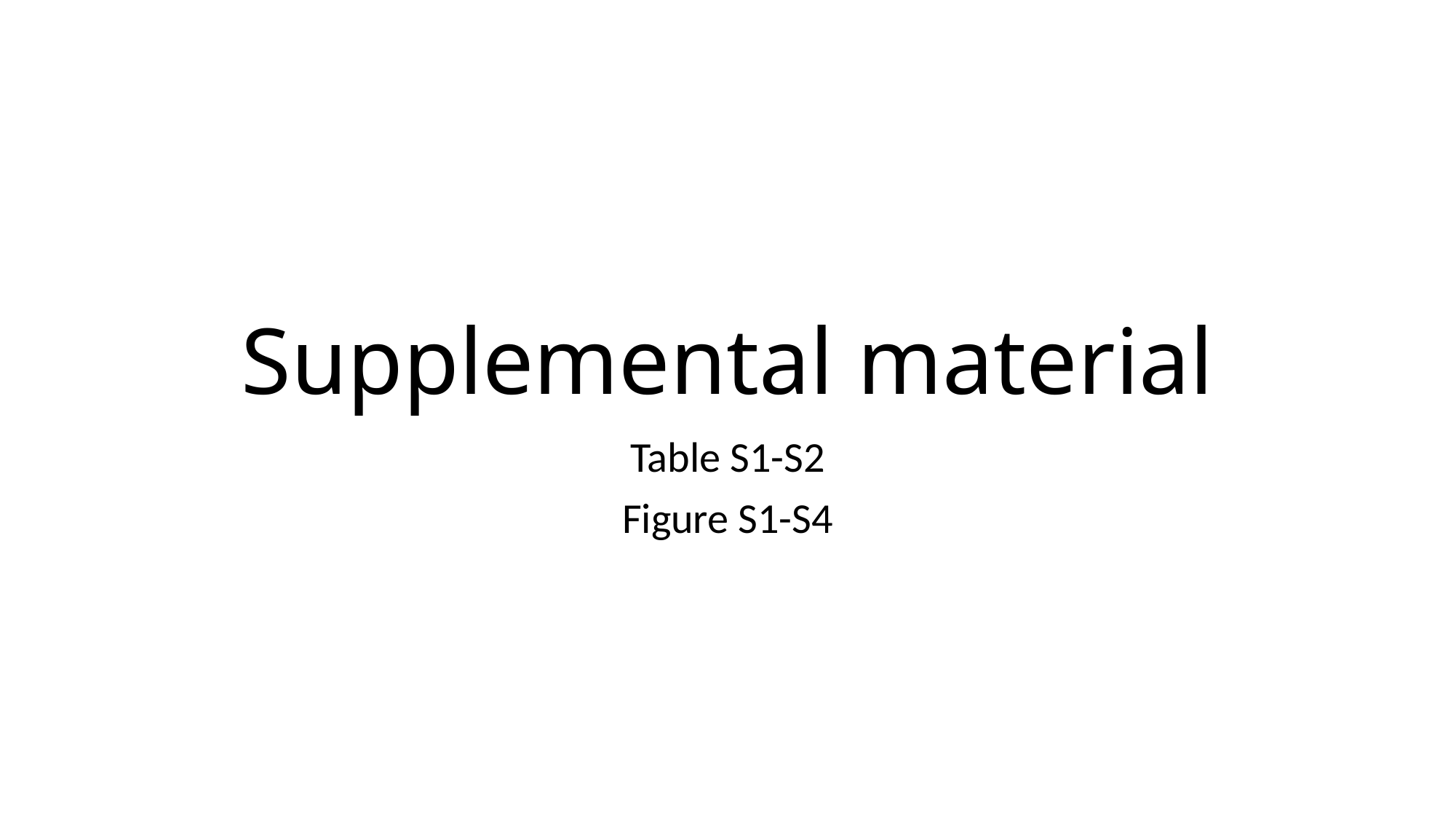

# Supplemental material
Table S1-S2
Figure S1-S4

## Slide 2
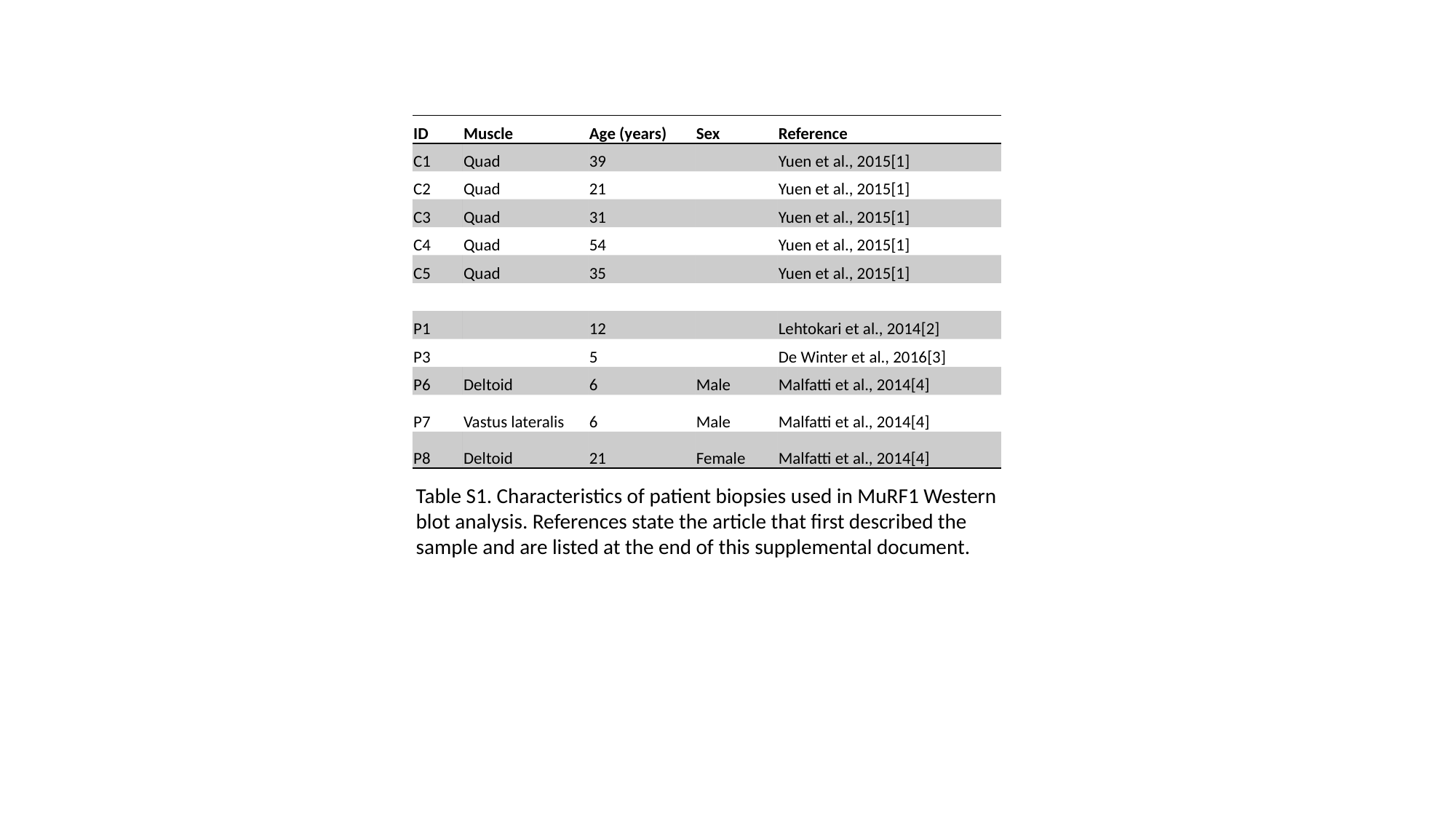

| ID | Muscle | Age (years) | Sex | Reference |
| --- | --- | --- | --- | --- |
| C1 | Quad | 39 | | Yuen et al., 2015[1] |
| C2 | Quad | 21 | | Yuen et al., 2015[1] |
| C3 | Quad | 31 | | Yuen et al., 2015[1] |
| C4 | Quad | 54 | | Yuen et al., 2015[1] |
| C5 | Quad | 35 | | Yuen et al., 2015[1] |
| | | | | |
| P1 | | 12 | | Lehtokari et al., 2014[2] |
| P3 | | 5 | | De Winter et al., 2016[3] |
| P6 | Deltoid | 6 | Male | Malfatti et al., 2014[4] |
| P7 | Vastus lateralis | 6 | Male | Malfatti et al., 2014[4] |
| P8 | Deltoid | 21 | Female | Malfatti et al., 2014[4] |
Table S1. Characteristics of patient biopsies used in MuRF1 Western blot analysis. References state the article that first described the sample and are listed at the end of this supplemental document.

## Slide 3
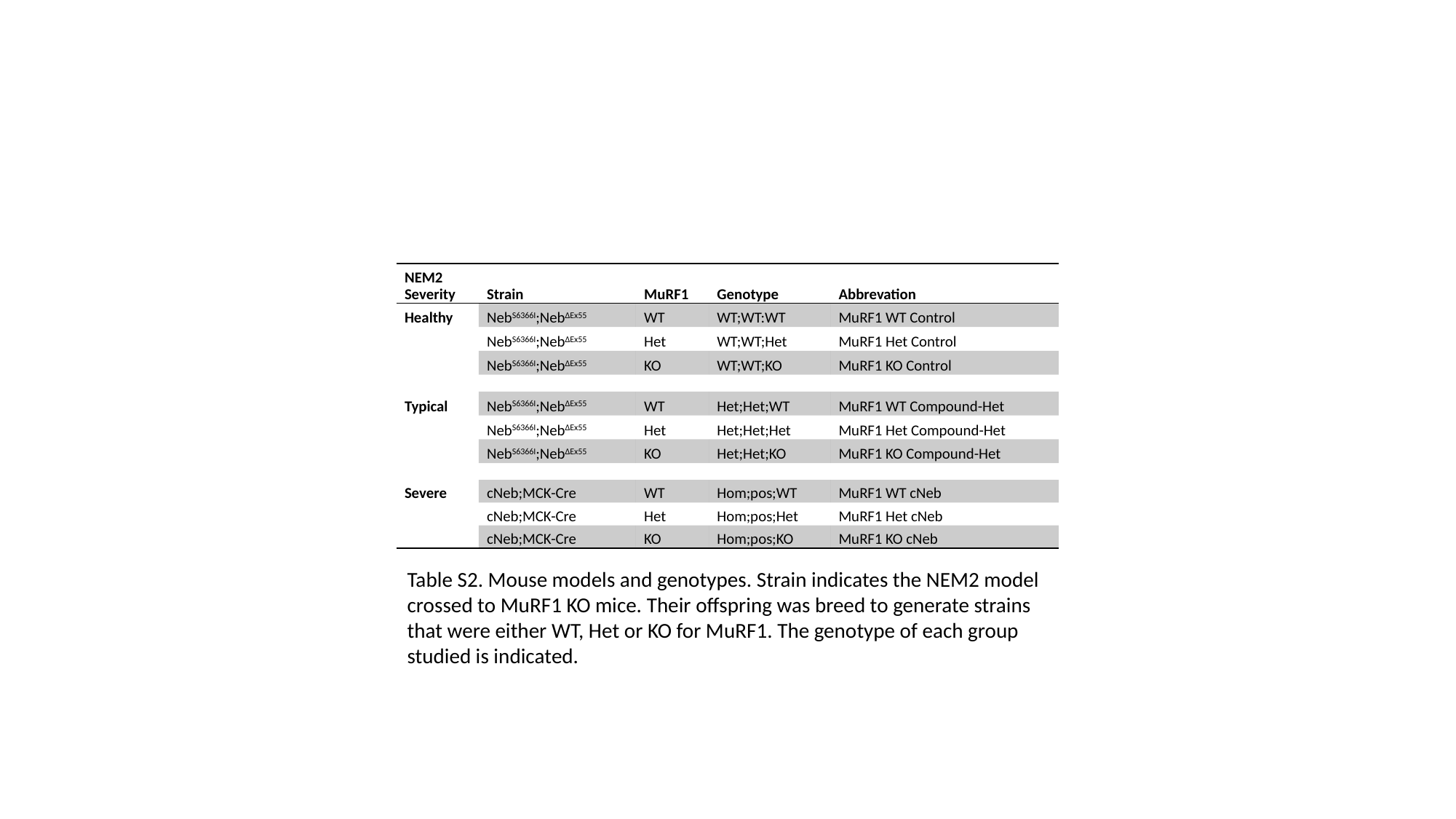

#
| NEM2 Severity | Strain | MuRF1 | Genotype | Abbrevation |
| --- | --- | --- | --- | --- |
| Healthy | NebS6366I;NebΔEx55 | WT | WT;WT:WT | MuRF1 WT Control |
| | NebS6366I;NebΔEx55 | Het | WT;WT;Het | MuRF1 Het Control |
| | NebS6366I;NebΔEx55 | KO | WT;WT;KO | MuRF1 KO Control |
| | | | | |
| Typical | NebS6366I;NebΔEx55 | WT | Het;Het;WT | MuRF1 WT Compound-Het |
| | NebS6366I;NebΔEx55 | Het | Het;Het;Het | MuRF1 Het Compound-Het |
| | NebS6366I;NebΔEx55 | KO | Het;Het;KO | MuRF1 KO Compound-Het |
| | | | | |
| Severe | cNeb;MCK-Cre | WT | Hom;pos;WT | MuRF1 WT cNeb |
| | cNeb;MCK-Cre | Het | Hom;pos;Het | MuRF1 Het cNeb |
| | cNeb;MCK-Cre | KO | Hom;pos;KO | MuRF1 KO cNeb |
Table S2. Mouse models and genotypes. Strain indicates the NEM2 model crossed to MuRF1 KO mice. Their offspring was breed to generate strains that were either WT, Het or KO for MuRF1. The genotype of each group studied is indicated.

## Slide 4
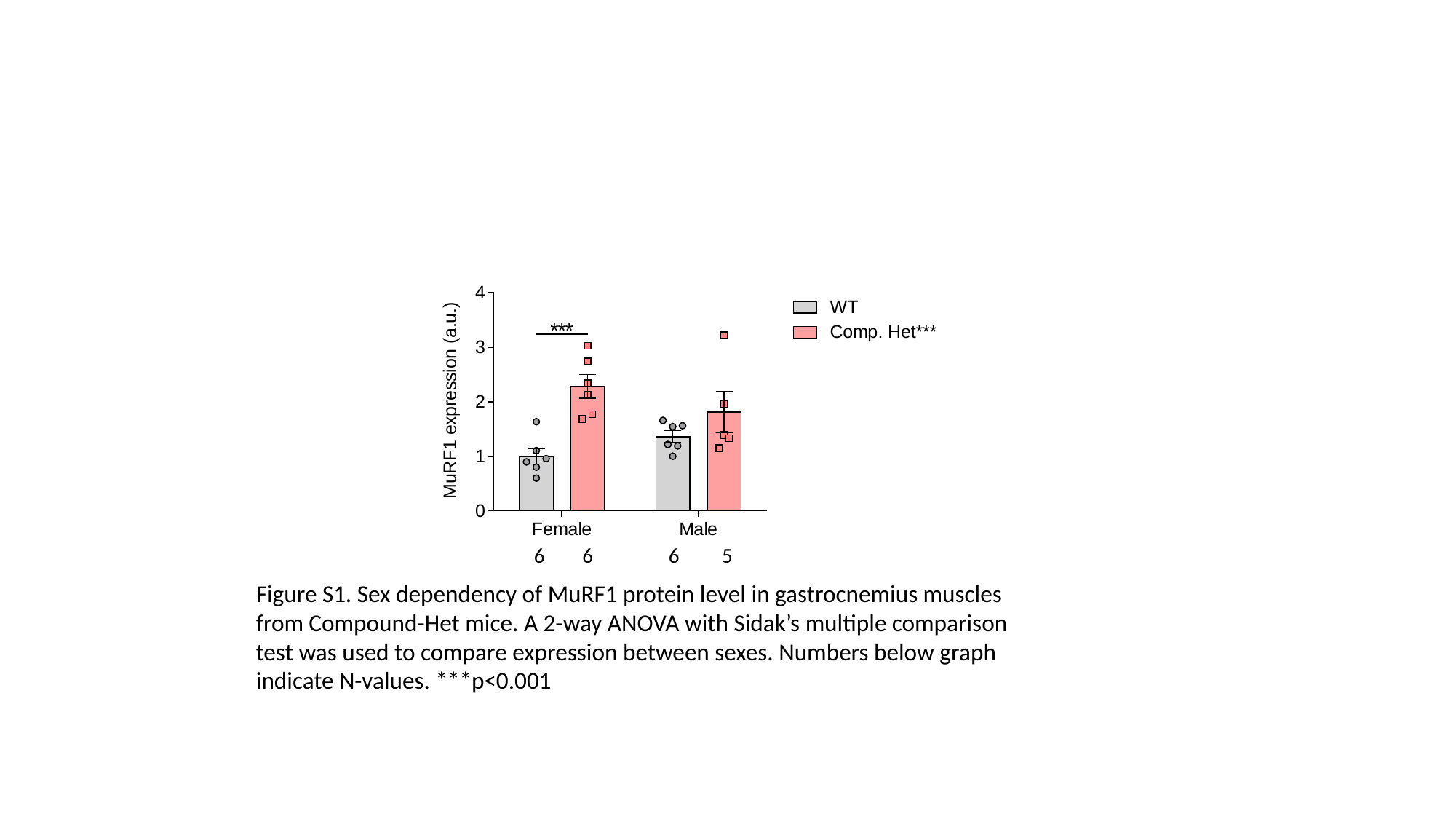

#
6 6 6 5
Figure S1. Sex dependency of MuRF1 protein level in gastrocnemius muscles from Compound-Het mice. A 2-way ANOVA with Sidak’s multiple comparison test was used to compare expression between sexes. Numbers below graph indicate N-values. ***p<0.001

## Slide 5
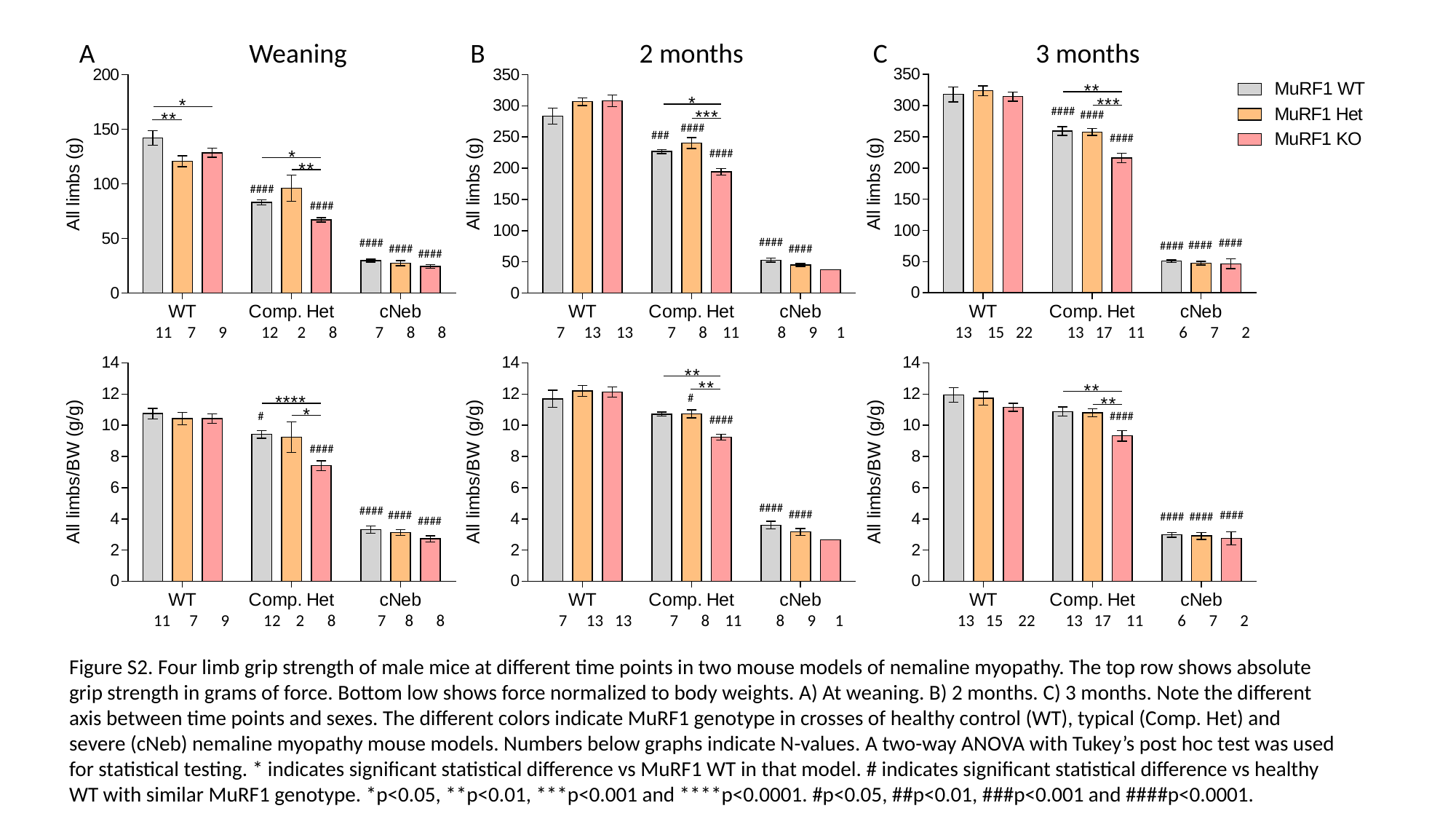

A Weaning B 2 months C 3 months
####
####
####
###
####
####
####
####
####
####
####
####
####
####
####
####
11 7 9 12 2 8 7 8 8 7 13 13 7 8 11 8 9 1 13 15 22 13 17 11 6 7 2
#
#
####
####
####
####
####
####
####
####
####
####
####
11 7 9 12 2 8 7 8 8 7 13 13 7 8 11 8 9 1 13 15 22 13 17 11 6 7 2
Figure S2. Four limb grip strength of male mice at different time points in two mouse models of nemaline myopathy. The top row shows absolute grip strength in grams of force. Bottom low shows force normalized to body weights. A) At weaning. B) 2 months. C) 3 months. Note the different axis between time points and sexes. The different colors indicate MuRF1 genotype in crosses of healthy control (WT), typical (Comp. Het) and severe (cNeb) nemaline myopathy mouse models. Numbers below graphs indicate N-values. A two-way ANOVA with Tukey’s post hoc test was used for statistical testing. * indicates significant statistical difference vs MuRF1 WT in that model. # indicates significant statistical difference vs healthy WT with similar MuRF1 genotype. *p<0.05, **p<0.01, ***p<0.001 and ****p<0.0001. #p<0.05, ##p<0.01, ###p<0.001 and ####p<0.0001.

## Slide 6
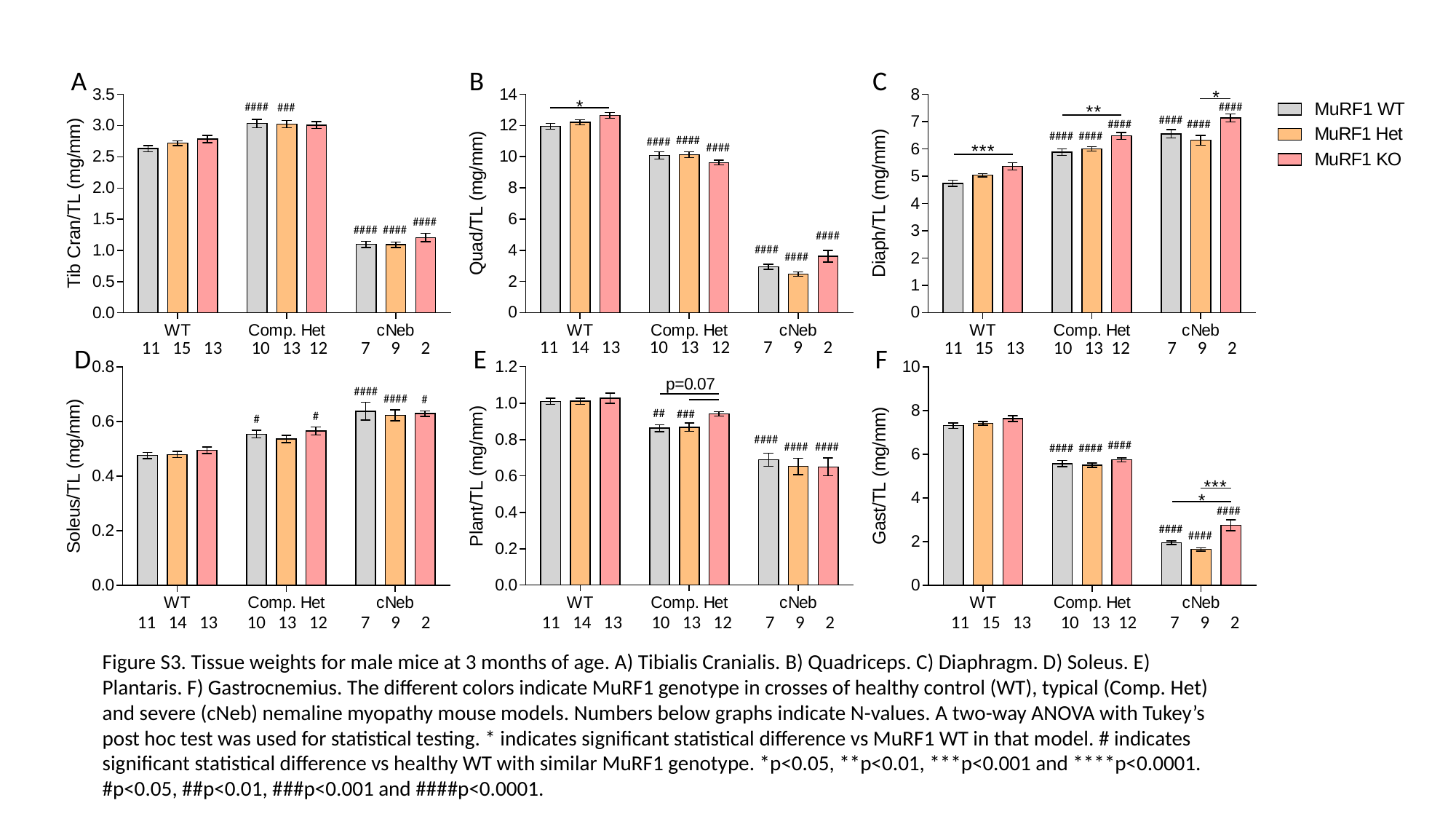

A B C
####
####
###
####
####
####
####
####
####
####
####
####
####
####
####
####
####
11 14 13 10 13 12 7 9 2
11 15 13 10 13 12 7 9 2
11 15 13 10 13 12 7 9 2
D E F
####
####
#
##
###
#
#
####
####
####
####
####
####
####
####
####
11 14 13 10 13 12 7 9 2
11 14 13 10 13 12 7 9 2
11 15 13 10 13 12 7 9 2
Figure S3. Tissue weights for male mice at 3 months of age. A) Tibialis Cranialis. B) Quadriceps. C) Diaphragm. D) Soleus. E) Plantaris. F) Gastrocnemius. The different colors indicate MuRF1 genotype in crosses of healthy control (WT), typical (Comp. Het) and severe (cNeb) nemaline myopathy mouse models. Numbers below graphs indicate N-values. A two-way ANOVA with Tukey’s post hoc test was used for statistical testing. * indicates significant statistical difference vs MuRF1 WT in that model. # indicates significant statistical difference vs healthy WT with similar MuRF1 genotype. *p<0.05, **p<0.01, ***p<0.001 and ****p<0.0001. #p<0.05, ##p<0.01, ###p<0.001 and ####p<0.0001.

## Slide 7
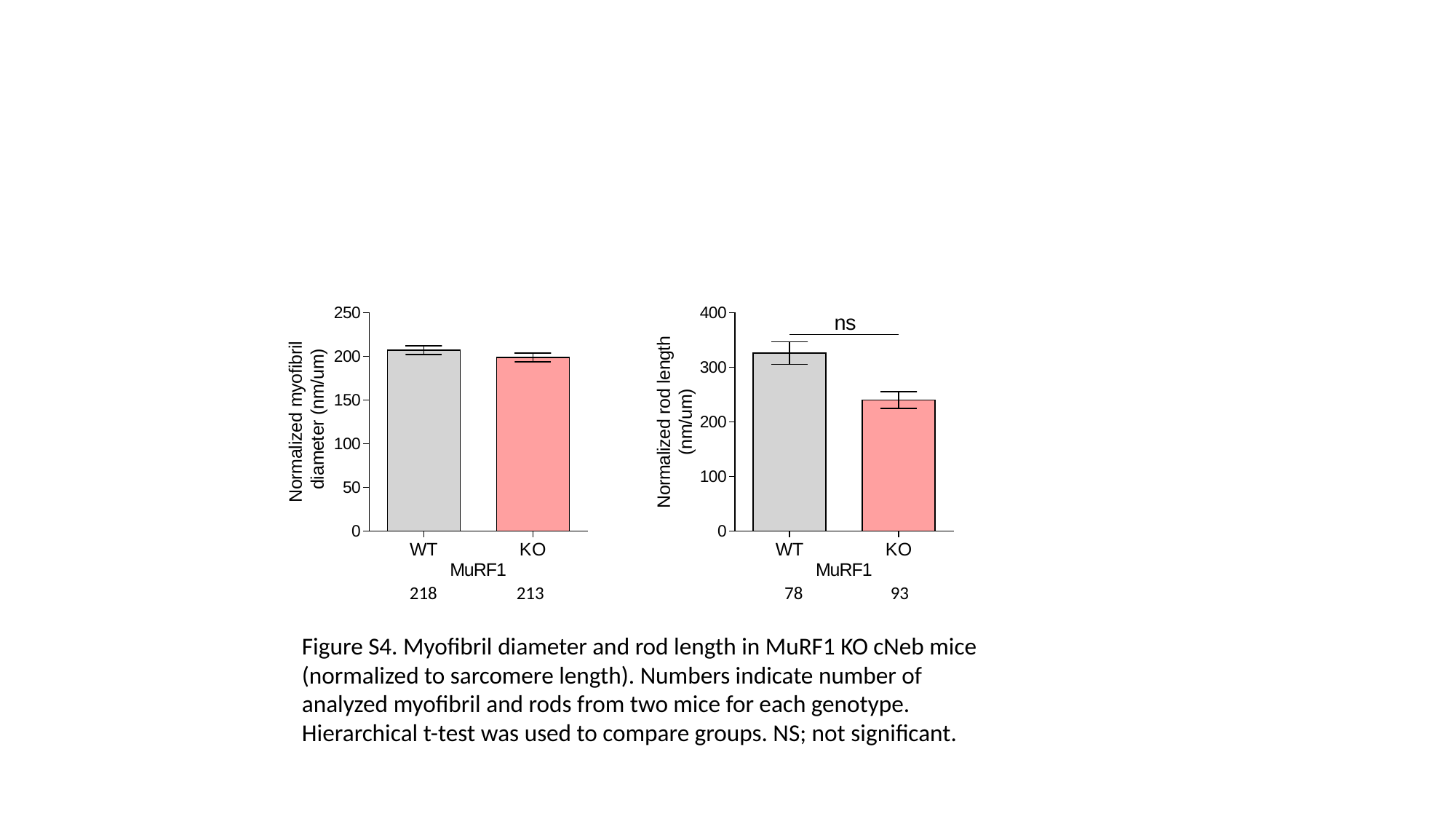

#
218 213
78 93
Figure S4. Myofibril diameter and rod length in MuRF1 KO cNeb mice (normalized to sarcomere length). Numbers indicate number of analyzed myofibril and rods from two mice for each genotype. Hierarchical t-test was used to compare groups. NS; not significant.

## Slide 8
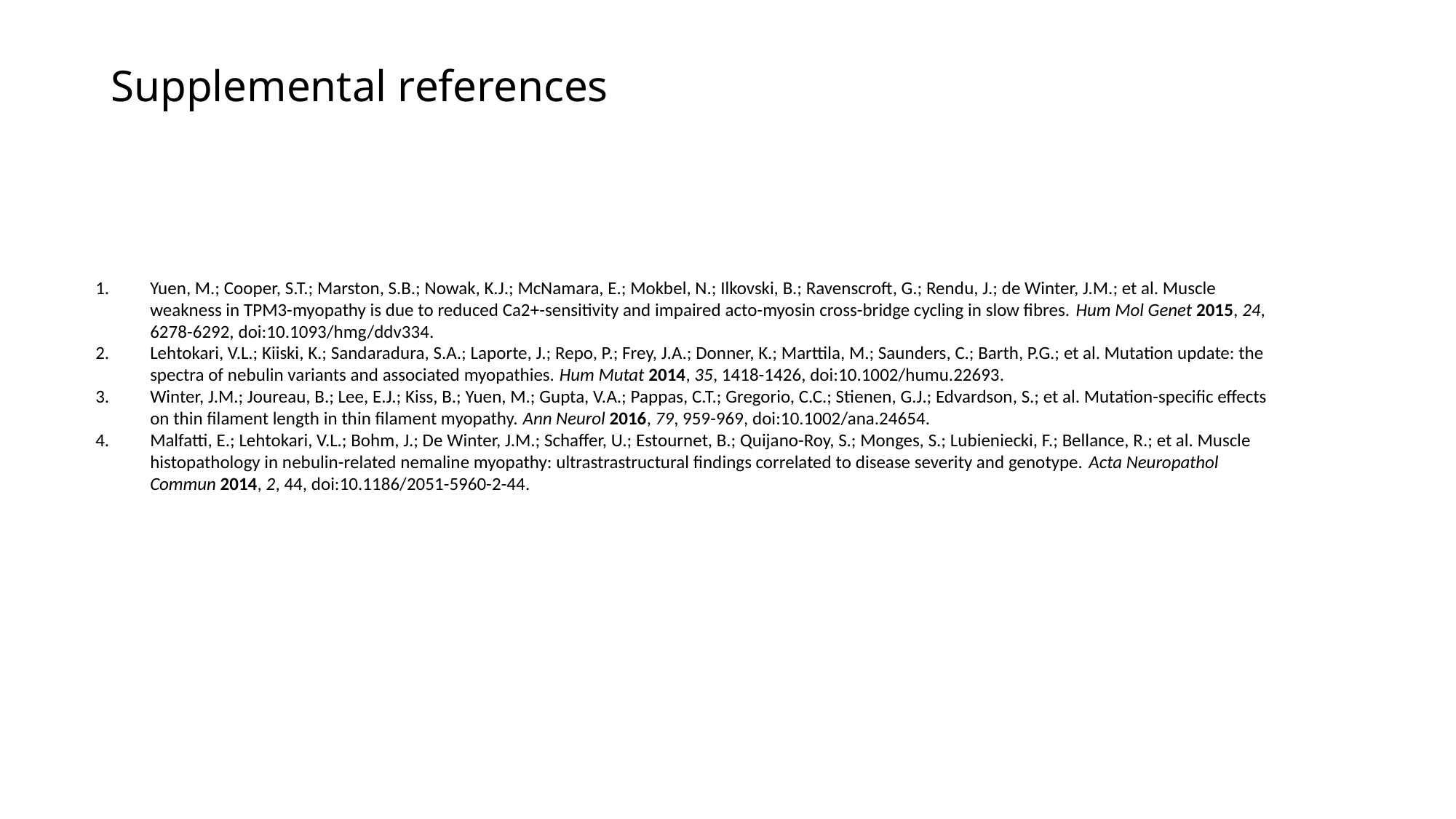

# Supplemental references
1.	Yuen, M.; Cooper, S.T.; Marston, S.B.; Nowak, K.J.; McNamara, E.; Mokbel, N.; Ilkovski, B.; Ravenscroft, G.; Rendu, J.; de Winter, J.M.; et al. Muscle weakness in TPM3-myopathy is due to reduced Ca2+-sensitivity and impaired acto-myosin cross-bridge cycling in slow fibres. Hum Mol Genet 2015, 24, 6278-6292, doi:10.1093/hmg/ddv334.
2.	Lehtokari, V.L.; Kiiski, K.; Sandaradura, S.A.; Laporte, J.; Repo, P.; Frey, J.A.; Donner, K.; Marttila, M.; Saunders, C.; Barth, P.G.; et al. Mutation update: the spectra of nebulin variants and associated myopathies. Hum Mutat 2014, 35, 1418-1426, doi:10.1002/humu.22693.
3.	Winter, J.M.; Joureau, B.; Lee, E.J.; Kiss, B.; Yuen, M.; Gupta, V.A.; Pappas, C.T.; Gregorio, C.C.; Stienen, G.J.; Edvardson, S.; et al. Mutation-specific effects on thin filament length in thin filament myopathy. Ann Neurol 2016, 79, 959-969, doi:10.1002/ana.24654.
4.	Malfatti, E.; Lehtokari, V.L.; Bohm, J.; De Winter, J.M.; Schaffer, U.; Estournet, B.; Quijano-Roy, S.; Monges, S.; Lubieniecki, F.; Bellance, R.; et al. Muscle histopathology in nebulin-related nemaline myopathy: ultrastrastructural findings correlated to disease severity and genotype. Acta Neuropathol Commun 2014, 2, 44, doi:10.1186/2051-5960-2-44.
